# Improved Blood Biomarkers but No Cognitive Effects from 16 Weeks of Multivitamin Supplementation in Healthy Older Adults

**DOI:** 10.3390/nu7053796

**Published:** 2015-05-19

**Authors:** Elizabeth Harris, Helen Macpherson, Andrew Pipingas

**Affiliations:** 1 Centre for Human Psychopharmacology, Swinburne University of Technology, Hawthorn, VIC 3122, Australia; E-Mails: eharris@swin.edu.au (E.H.); apipingas@swin.edu.au (A.P.); 2 Centre for Physical Activity and Nutrition Research, School of Exercise and Nutrition Sciences, Deakin University, Burwood, VIC 3125, Australia

**Keywords:** multivitamins, vitamins, cognition, biomarkers

## Abstract

Supplementation with vitamins, minerals and phytonutrients may be beneficial for cognition, especially in older adults. The aim of this study was to assess the effects of multivitamin supplementation in older adults on cognitive function and associated blood biomarkers. In a randomised, double blind, placebo-controlled trial, healthy women (*n* = 68) and men (*n* = 48) aged 55–65 years were supplemented daily for 16 weeks with women’s and men’s formula multivitamin supplements. Assessments at baseline and post-supplementation included computerised cognitive tasks and blood biomarkers relevant to cognitive aging. No cognitive improvements were observed after supplementation with either formula; however, several significant improvements were observed in blood biomarkers including increased levels of vitamins B_6_ and B_12_ in women and men; reduced *C*-reactive protein in women; reduced homocysteine and marginally reduced oxidative stress in men; as well as improvements to the lipid profile in men. In healthy older people, multivitamin supplementation improved a number of blood biomarkers that are relevant to cognition, but these biomarker changes were not accompanied by improved cognitive function.

## 1. Introduction

Poor nutrition is a risk factor for cognitive decline in old age [1], which suggests that addressing nutritional insufficiencies may improve cognitive outcomes. The various essential vitamins and minerals that are required in the diet to maintain general health also have many functions in the brain. These include roles in neuroprotection, neurotransmission, homeostatic regulation, antioxidant activities, energy metabolism and DNA synthesis [2]. In addition, various plant extracts have purported benefits to cognitive function, including *Ginkgo biloba* and *Bacopa monnieri* [3,4].

Minimum daily requirements for vitamins and minerals have been set by government and other organisations around the world [5]. However, individual requirements differ and the level required for sufficient reserves in the body and maintenance of optimal metabolic activity is unknown [6]. This is particularly true for psychological function including cognitive abilities such as memory and attention [7]. Providing nutrients at levels above the recommended intake might therefore be beneficial, especially for vulnerable groups. Older adults are at increased risk of nutritional deficiency for vitamins including folate, vitamin B_6_, vitamin B_12_, and vitamin D; and minerals including zinc, calcium, magnesium; as well as decreased intake of phytonutrients [8].

Cognitive decline has been associated with changes in various biomarkers, some of which have been demonstrated to be sensitive to supplementation with nutraceuticals. Of particular interest are biomarkers relevant to nutritional status, which can be used to identify potential pathological processes, which contribute to cognitive decline. For example, high homocysteine is associated with dementia and poorer cognition, and is reduced by supplementation with B-group vitamins, particularly folate and B_12_ [9,10,11]. The inflammatory response is also associated with poorer cognition in later life, and increased inflammatory biomarkers are present in dementia [12,13]. Many nutrients are purported to have anti-inflammatory actions, including quercetin [14], vitamin D [15], and vitamin B_6_ [16,17]. Oxidative stress has been extensively studied in relation to cognitive health, with biomarkers of oxidative stress observed to be increased in people with cognitive decline and dementia [18]. Biomarkers of oxidative stress can be reduced with antioxidant vitamins [19] and plant extracts including flavonoids [20].

Epidemiological data have provided numerous associations between nutritional factors, associated biomarkers, and cognitive decline or dementia [21]. Intervention studies aiming to improve cognitive outcomes with supplements have had varying success, with some [22,23,24,25], but not all interventions yielding cognitive benefits [26,27]. Differences in supplement dose, duration and participant characteristics may be responsible for inconsistent findings. It is possible that nutritional supplements may exert maximal benefit prior to the onset of cognitive decline by addressing subtle nutritional deficiencies. Therefore, in supplement trials it may be useful to assess measures of nutritional status that also represent biomarkers of longer term poorer cognitive outcomes, especially those which may play an etiological role.

Secondly, it might be advantageous to use a broad range of nutrients such as in a multivitamin. It is plausible that combinations of nutrients may produce stronger effects because they have the capacity to address diverse nutritional requirements or deficiencies that might occur between individuals. Combination supplements also ensure that the benefits of a given nutrient are not limited by shortage of another; for example, both folate and B_12_ are involved in homocysteine metabolism; and vitamin C can ‘recycle’ or ‘refresh’ vitamin E [6,28].

Thirdly, it is imperative that sensitive tests are selected. Tests such as the Mini Mental State Examination (MMSE) or the Telephone Interview for Cognitive Status (TICS), which are frequently used in cognitive research, might not be precise enough to pick up subtle changes in supplement studies [23,27]. Computerized tests that assess faculties known to decline with age would be a better choice due to millisecond resolution and standardization of presentation. For example, the computerized cognitive battery used in our previous research has been demonstrated to be sensitive to age and nutritional supplementation [22,29].

The present study aimed to address these methodological issues in a randomized, controlled trial of two multivitamin supplements in older adults. The primary outcome measures for cognition were performance on computerized cognitive tasks, in particular the Spatial Working Memory, Contextual Memory and Stroop Interference tasks of the computerized battery. These tasks assess abilities that are most susceptible to cognitive decline, including spatial functions, episodic memory, and inhibition/executive function [29] and performance on these tasks has been demonstrated to improve after nutritional supplementation including multivitamins [22,30] and flavonoids [31]. The remaining cognitive tasks were included as secondary outcome measures.

Blood biomarkers were also assessed to provide information on the physiological effects of the supplement and to provide information regarding possible mechanisms of action. Blood biomarkers were considered to be secondary outcome measures.

## 2. Method

The study involved two randomised, double-blind, placebo-controlled trials, which investigated the effects of 16-weeks supplementation with two multivitamin, mineral and herbal supplements: a Women’s formula and a Men’s formula in women and men, respectively. The study was registered with the Australian New Zealand Clinical Trials Registry, trial ID ACTRN12608000117314. Data collection was carried out from August 2007 to December 2008. Ethics approval for the study was obtained from the Swinburne University Human Research Ethics Committee.

### 2.1. Participants

Participants were older adults aged 55 to 65 years, including 68 women and 48 men, who were healthy and free from any cognitive disorder. They were recruited via advertisements in local newspapers and seniors’ publications, noticeboards, or by word-of-mouth. Exclusion criteria included: smoking; use of multivitamins or combinations of vitamins or herbal supplements; self-reported history of psychological or neurological disorders, heart disease or stroke; and those taking blood-thinning medication (except if less than 100 mg aspirin). Volunteers were initially screened for the exclusion criteria via telephone and again on attendance at the baseline testing session. In addition, the MMSE was conducted at baseline, with a cut-off set at 27 from a possible score of 30 to determine eligibility into the study. All participants gave written informed consent.

### 2.2. Multivitamin Supplements and Placebo

The supplements used in the study were Swisse Women’s Ultivite Multi-Vitamin, Mineral & Anti-Oxidant with Herbs 50+ Years™ (Women’s formula) and Swisse Men’s Ultivite Multi-Vitamin, Mineral & Anti-Oxidant with Herbs 50+ Years™ (Men’s formula). These multivitamins are widely available in Australia. Both formulas contained vitamins, minerals, plant extracts and probiotics, however they differed in the dosages of vitamins and there were differences in some herbal ingredients, which have gender-specific functions. Of particular note, only the Women’s formula contained *Bacopa monnieri*, which has putative cognition-enhancing functions [4]. A full list of ingredients is provided in Table 1. The placebo tablets contained starch with a small amount of vitamin B_2_ (riboflavin) to produce a similar smell and some colouration of the urine. Active and placebo tablets were identical in colour (Women’s formula were dark pink, Men’s formula were green) and were the same size and shape. Tablets were provided in blister packs; used blister packs were returned and tablets counted to determine compliance.

**Table 1:** Ingredients of Swisse Ultivite 50+ Women’s & Men’s Formulas and Reference Daily Intakes (RDI) where applicable.

Ingredient	Women’s	RDI Women	Men’s	RDI Men
Retinyl Acetate	862.5 μg	700 μg	862.5 μg	900 μg
d-Alpha-Tocopheryl Acid Succinate	20 mg	7 mg	25 mg	10 mg
Thiamine Hydrochloride (vitamin B_1_)	30 mg	1.1 mg	35 mg	1.2 mg
Riboflavine (vitamin B_2_)	30 mg	1.1 mg	35 mg	1.3 mg
Nicotinamide (vitamin B_3_)	20 mg	14 mg	25 mg	16 mg
Calcium Pantothenate (vitamin B_5_)	70 mg	4 mg	75 mg	6 mg
Pyrodoxine Hydrochloride (vitamin B_6_)	30 mg	1.5 mg	25 mg	1.7 mg
Cyanocobalamin (vitamin B_12_)	115 μg	2.4 μg	120 μg	2.4 μg
Cholecalciferol (vitamin D_3_)	5 μg	10 μg	5 μg	10 μg
Biotin (vitamin H)	150 μg	25 μg	200 μg	30 μg
Folic Acid	500 μg	400 μg	500 μg	400 μg
Calcium Ascorbate Dihydrate (vitamin C)	200 mg	45 mg	200 mg	45 mg
Phytomedadione (vitamin K_1_)	60 μg	60 μg	70 μg	70 μg
Calcium Orotate	100 mg	1000 mg	100 mg	1300 mg
Magnesium Aspartate Dihydrate	100 mg	320 mg	100 mg	420 mg
Selenomethionine	65 μg	60 μg	65 μg	70 μg
Molybdenum Trioxide	67.5 μg	45 μg	67.5 μg	45 μg
Chromium Picolinate	402 μg	25 μg *	402 μg	35 μg *
Manganese Amino Acid Chelate	30 mg	5 mg	40 mg	5.5 mg
Ferrous Fumarate	16.01 mg	8 mg	16.01 mg	8 mg
Copper Gluconate	8.57 mg	1.2 mg *	12.14 mg	1.7 mg *
Potassium Iodide	196 μg	150 μg 2800 mg *	196 μg	150 μg 3800 mg *
Zinc Amino Acid Chelate	75 mg	8 mg	100 mg	14 mg
**Ingredient**	**Women’s**	****	**Men’s**	
Lactobacillus rhamnosus	80 million organisms		80 million organisms	
Lactobacillus acidophilus	80 million organisms		80 million organisms	
Bifidobacterium longum	35 million organisms		35 million organisms	
Vaccinium Macrocarpon Fruit Dry (patented cranberry PACRAN)	800 mg		1000 mg	
Citrus Bioflavoloids Extract	20 mg		20 mg	
Silybum Marianum Dry Fruit (St. Mary’s thistle)	1500 mg		1700 mg	
Ginkgo Biloba Leaf Dry	1000 mg		1000 mg	
Crataegus Monogyna Fruit Dry (Hawthorn)	100 mg		120 mg	
Cynara Scolymus Leaf Dry (Globe artichoke)	50 mg		50 mg	
Lecithin Powder—Soy Phosphatidylserine Enriched Soy	10 mg		10 mg	
Scutellaria Lateriflora Herb Dry (Skullcap)	50 mg		50 mg	
Spearmint Oil	2 mg		2 mg	
Tagetes Erecta Flower Dry (Marigold)	100 mg		100 mg	
Ubidecarenone (Co-enzyme Q10) (from patented Ultrasome CoQ10)	2 mg		3 mg	
Urtica Dioica Leaf Dry (Nettle)	100 mg		50 mg	
Vaccinium Myrtillus Fruit Dry (Bilberry)	100 mg		100 mg	
Vitis Vinifera Dry Seed (Grape seed)	1000 mg		1000 mg	
Bacopa Monnieri Whole Plant Dry	50 mg		-	
Cimifuga Racemosa Root & Rhizome Dry (Black cohosh)	200 mg		-	
Curcuma Longa Rhizome Dry (Turmeric)	100 mg		-	
Silica Colloidal Anhydrous	20 mg		-	
Tunera Diffusa Leaf Dry (Damiana)	500 mg		-	
Withania Somnifera Root Dry (Ashwagandha)	500 mg		-	
Dulacia Inopiflora Root Dry (Muirapuama)	-		200 mg	
Serenoa Repens Fruit Dry (Saw palmetto)	-		300 mg	
Tribulus Terrestris Fruit & Root Dry (Tribulus)	-		1000 mg	

* Adequate intake where RDI values were not available.

### 2.3. Sample Size Calculation and Randomisation

Two previous studies conducted by our group, which tested nutraceutical products and used the same cognitive test battery, obtained moderate effect sizes, with eta-squared (η^2^) values of 0.484 [22] and 0.314 [30]. G*Power software 3.1.7 [32] was used to calculate the required sample size, using an effect size of 0.4, alpha of 0.05 and power of 0.80. It was determined that 52 participants were required for each gender for the study to have sufficient power to detect a moderate effect size. Randomisation was performed by Swisse Wellness Pty Ltd. (Melbourne, Vic, Australia) and was conducted in blocks of four with an allocation ratio of 1:1. Multivitamin and placebo tablets were provided by Swisse Wellness Pty Ltd. and given to the investigator in numbered packets according to the allocation schedule. Swisse Wellness Pty Ltd. held the allocation schedule until data collection was completed, thus all investigators were blind to allocation for the duration of data collection. Numbers were allocated sequentially as participants were enrolled in the study.

### 2.4. Measures and Procedures

Participants in both trials followed the same protocol. They were assessed on a range of demographic measures, cognitive performance tasks, blood biomarkers, and cardiovascular measures (cardiovascular results to be reported separately). Demographic measures included date of birth, number of years of education, body mass index, and details of medication or supplement use.

Cognitive testing comprised two equivalent versions of a battery of eight computerized cognitive tests, which took approximately 45 min to complete. Responses were made on a response box with four coloured buttons arranged in the positions of compass poles as follows: top/yellow, bottom/green, left/blue/no, and right/red/yes. The tasks included in the battery were: Simple Reaction Time (fast response to presentation of a white square); Choice Reaction Time (blue/red response to a blue triangle or red square), Immediate and Delayed Recognition Memory (for abstract images, yes/no response), Stroop Congruent (red/yellow/green/blue response to the colour of the word, with matching text), Stroop Incongruent (red/yellow/green/blue response to colour of the word, with non-matching text), Spatial Working Memory (yes/no response to location of white squares in a grid) and Contextual Memory (top/bottom/left/right response to indicate original location of previously presented everyday items). The cognitive battery has been described in detail elsewhere [22].

Blood biomarkers were assessed using a commercial pathology company. Tests included whole blood pyridoxal 5’-phosphate (vitamin B_6_); serum vitamin B_12_; red cell folate; serum homocysteine; serum high-sensitivity *C*-reactive protein (CRP); whole blood fibrinogen; plasma protein carbonyls; full lipid profile including total cholesterol, HDL, LDL and triglycerides; kidney and liver function tests including aspartate transaminase (AST) and alanine transaminase (ALT). All tests were performed from fresh samples, except protein carbonyls, which were frozen.

Participants attended two testing sessions (baseline and post-treatment) held in laboratories optimised for the collection of psychometric data at Swinburne University of Technology. The duration of the sessions was 1.5 h and participants were asked to refrain from ingesting caffeine for three hours beforehand. Testing sessions were conducted for each participant individually in the following order: informed consent, demographic information, MMSE, blood pressures, height and weight, and computerized cognitive tasks. Upon finishing testing, the participants were provided with a referral for blood testing and instructions to fast overnight and attend the blood collection centre early in the morning the following day or as early as possible thereafter. Participants began supplementation on the day following blood testing and did not take supplements on return testing days, ensuring that only chronic effects of the supplements were measured, that is, acute effects were not observed.

### 2.5. Statistical Analysis

Data analysis was performed using IBM SPSS 20. Women’s and Men’s groups were analysed separately. Univariate analysis of covariance (ANCOVA) was used to determine the effects of multivitamin supplementation on cognitive task performance and blood biomarkers. For each test, the post-treatment result was entered as the dependent variable, the baseline result entered as the covariate, and treatment group was entered as a fixed factor.

Data was initially screened to ensure it met the assumptions for ANCOVA including normality and homogeneity of variance. The assumptions were met except for the distribution of CRP, which was highly positively skewed. A logarithmic transformation was applied (log10^(x)^), which improved the distribution. To avoid undue distortion of the data, values more than three standard deviations from the mean were not included in the analysis 

Significance levels were set at *p* < 0.05 however results with significance levels *p* < 0.10 are also noted, to acknowledge any non-significant trends in the data. There were three primary outcome measures (Spatial Working Memory, Contextual Memory and Stroop Interference tasks) and adjustments were made to correct for multiple comparisons. Significance was calculated as 0.05/3 = 0.016.

## 3. Results

**Figure 1:**
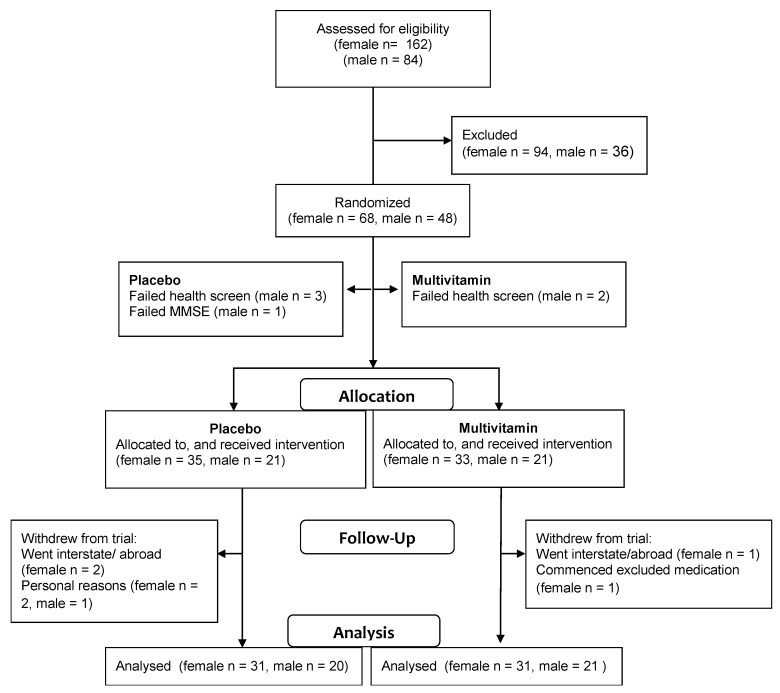
Flowchart of participant involvement.

Participant numbers at recruitment, randomisation and follow up are shown in Figure 1. The multivitamin and placebo groups were well matched on demographic variables as shown in Table 2. Independent *t*-tests indicated that there were no significant differences between the group means for age, years of education, or body mass index (BMI) at baseline (*p* > 0.05). Mean compliance was 95.8% for women and men, which was confirmed by counting remaining tablets at follow up.

**Table 2:** Participant characteristics at baseline.

Characteristic	Placebo Multivitamin				
	*M (SD)*	Range	*M (SD)*	Range	*p*
**Males**					
Age—*years*	59.1 (2.3)	55–65	60.2 (3.2)	55–65	0.214
Education—*years*	16.5 (3.8)	11–27	16.0 (3.9)	11–23	0.602
BMI—*kg/m^2^*	26.8 (2.9)	21.4–32.3	26.8 (6.2)	19.0–49.2	0.138
**Females**					
Age—*years*	60.1 (3.4)	54–65	60.2 (3.2)	55–65	0.818
Education—*years*	14.7 (3.8)	9–24	16.0 (3.9)	11–23	0.163
BMI—*kg/m^2^*	26.3 (4.4)	18.5–36.5	26.8 (6.2)	19.0–49.1	0.717

*p*-Values are shown for independent groups *t*-tests.

### 3.1. Cognitive Test Results—Women’s and Men’s Formulas

Mean accuracy and response time scores for the cognitive tests are presented in Table 3. ANCOVAs revealed no effect of supplementation on cognitive task performance for the primary outcome measures, Spatial Working Memory, Contextual Memory and Stroop Interference. Therefore the hypothesis that these tasks would improve with multivitamin supplementation was not supported. There were no significant effects observed on any of the other cognitive measures. A trend for a treatment effect was observed for Choice Response Time in the Men’s group (*p* = 0.053), however this was not corrected for multiple comparisons and may be a chance finding.

### 3.2. Blood Test Results—Women’s Formula

Blood tests results for baseline and post-treatment are presented in Table 4. There was a significant treatment effect on vitamin B_6_ (*F*(1,48) = 55.5, *p* < 0.001 partial η^2^ = 0.536), with substantial increases in vitamin B_6_ for women supplemented with the multivitamin, compared with only a very small change in those taking placebo. There was also a significant treatment effect for vitamin B_12_ (*F*(1,56) = 30.5, *p* < 0.001 partial η^2^ = 0.352), with an increase in B_12_ in the multivitamin group compared with no change in the placebo group.

**Table 3:** Cognitive tests: Means and standard deviations for accuracy (*%*) and response time (*ms*). No significant effects of supplementation were observed.

		Females			Males		
		Baseline	Post	*n*	Baseline	Post	*n*
		*M (SD)*	*M (SD)*		*M (SD)*	*M (SD)*	
Spatial Mem., *%*	PL	67.1 (13.8)	70.5 (10.6)	30	78.1 (9.9)	81.5 (9.2)	16
	MV	68.7 (13.2)	71.4 (13.3)	26	74.9 (16.1)	77.0 (15.7)	21
Spatial Mem., *ms*	PL	1200 (190)	1132 (127)	30	1023 (136)	996 (115)	16
	MV	1146 (166)	1099 (161)	26	1018 (181)	975 (183)	21
Contextual, *%*	PL	74.3 (17)	73.2 (11.7)	28	69.7 (13.0)	63.8 (12.5)	16
	MV	78.5 (13)	71.1 (13.0)	27	73.4 (17.7)	64.2 (16.9)	19
Contextual, *ms*	PL	1130 (157)	1081 (135)	29	1069 (131)	1082 (116)	16
	MV	1070 (123)	1073 (139)	27	1077 (135)	1061 (168)	19
Stroop Interfere., *ms*	PL	205 (134)	217 (142)	27	220 (207)	152 (109)	15
	MV	187 (135)	188 (139)	23	121 (94)	132 (80)	17
Stroop Congruent, *ms*	PL	775 (91.6)	784 (88)	29	746 (81)	780 (95)	16
	MV	776 (109)	745 (93)	27	743 (99)	726 (102)	21
Stroop Incong., *%*	PL	94.1 (6.2)	93.7 (9.7)	28	91.5 (15.3)	93.2 (7.6)	15
	MV	94.0 (11.4)	94.9 (6.0)	23	97.8 (3.4)	97.5 (4.2)	17
Stroop Incong., *ms*	PL	979 (148)	1000 (140)	28	970 (165)	938 (106)	15
	MV	941 (185)	919 (177)	23	851 (127)	854 (99)	17
Simple RT, *ms*	PL	278 (27.1)	274 (33)	27	262 (32)	257 (27)	17
	MV	276 (26.8)	272 (29)	25	260 (24)	258 (17)	20
Choice RT, *ms*	PL	491 (61)	485 (58)	27	462 (42)	488 (63)	17
	MV	499 (66)	501 (65)	27	467 (56)	457 (50)	20
Immed. Recog., *%*	PL	67.4 (11.7)	76.4 (9.0)	28	70.7 (12.0)	79.8 (7.6)	15
	MV	70.3 (13.0)	78.2 (8.0)	24	67.0 (12.2)	76.7 (8.7)	20
Immed. Recog., *ms*	PL	1151 (161)	1087 (133)	28	1129 (174)	1095 (160)	15
	MV	1084 (102)	1054 (127)	24	1144 (153)	1060 (179)	20
Delayed Recog., *%*	PL	68.0 (8.7)	75.2 (10.4)	28	66.0 (14.3)	70.5 (7.6)	14
	MV	71.2 (9.4)	73.6 (12.3)	26	69.0 (10.0)	70.7 (14.0)	20
Delayed Recog., *ms*	PL	1064 (141)	1020 (126)	28	1123 (162)	1081 (155)	14
	MV	1077 (128)	1023 (148)	26	1073 (95)	1014 (147)	19

MV = multivitamin, PL = placebo.

There was a significant treatment effect for homocysteine (*F*(1,56) = 6.73, *p* = 0.012, partial η^2^ = 0.107), however *post-hoc* *t*-tests indicated that there was increased homocysteine in the placebo group (*t*(30) = −2.65, *p* = 0.013) rather than a decrease in the multivitamin group, which was not significant (*t*(27) = 0.705, *p =* 0.487).

Analysis of the transformed data for CRP revealed a reduction with multivitamin supplementation compared with placebo (*F*(1,51) = 8.60, *p* = 0.005, partial η^2^ = 0.152). There were no significant treatment effects observed for fibrinogen, protein carbonyls, or for any of the lipid measures in the Women’s group.

**Table 4:** Blood test results for women and men at baseline and post-treatment for placebo and multivitamin groups.

Test *Units*		Females			Males		
		Baseline	Post-Treatment	*n*	Baseline	Post-Treatment	*n*
		*M (SD)*	*M (SD)*		*M (SD)*	*M (SD)*	
Homocysteine, µ*mol/L*	PL	13.0 (2.9)	14.4 (3.9)	31 ^‡^	15.5 (3.9)	16.2 (3.2)	17 ^†^
	MV	12.9 (3.0)	12.6 (2.5)	28	15.1 (3.4)	13.7 (2.8)	21
Vitamin B_12_, *pmol/L*	PL	300 (78)	298 (91)	31 *	284 (100)	249 (84)	15 *
	MV	300 (103)	435 (143)	28	265 (63)	342 (120)	19
Vitamin B_6_, *nmol/L*	PL	88 (28)	118 (92)	28 *	151 (120)	160 (155)	13 *
	MV	104 (53)	467 (215)	23	121 (89)	335 (134)	15
Folate, *nmol/L*	PL	744 (290)	800 (228)	30	601 (187)	614 (156)	17
	MV	922 (180)	933 (309)	28	683 (298)	793 (303)	19
CRP, *mg/L*	PL	1.9 (2.2)	1.8 (2.1)	27 ^†^	1.4 (1.5)	0.9 (0.9)	15
	MV	1.9 (2.5)	1.1 (1.2)	24	1.9 (1.7)	1.1 (0.8)	20
Fibrinogen, *g/L*	PL	3.1 (0.3)	3.0 (0.4)	30	3.0 (0.6)	3.1 (0.3)	14
	MV	3.0 (0.5)	3.0 (0.5)	26	2.9 (0.4)	2.9 (0.5)	20
Prot Carbonyl, *mol/mL*	PL	19.6 (7.1)	17.6 (3.5)	24	19.1 (5.2)	19.4 (4.5)	15
	MV	18.2 (7.05)	19.0 (5.1)	24	20.5 (5.1)	16.4 (4.6)	15
Total Chol, *mmol/L*	PL	6.0 (1.1)	5.8 (0.9)	31	5.6 (0.9)	5.7 (1.1)	17
	MV	5.7 (0.8)	5.8 (0.8)	29	5.6 (0.7)	5.3 (0.9)	21
HDL, *mmol/L*	PL	1.80 (0.40)	1.88 (0.48)	31	1.54 (0.30)	1.52 (0.36)	17
	MV	1.90 (0.38)	2.07 (0.56)	29	1.51 (0.42)	1.46 (0.39)	20
LDL, *mmol/L*	PL	3.70 (0.97)	3.40 (1.05)	31	3.42 (0.67)	3.44 (0.79)	17 ^‡^
	MV	3.37 (0.66)	3.11 (0.96)	29	3.45 (0.59)	3.06 (0.89)	20
Triglyceride, *mmol/L*	PL	1.2 (0.5)	1.2 (0.6)	31	1.2 (0.5)	1.4 (0.6)	16
	MV	1.0 (0.4)	1.1 (0.4)	28	1.3 (0.5)	1.4 (0.6)	21
LDL/HDL ratio	PL	2.2 (0.7)	2.1 (0.7)	31	2.3 (0.6)	2.2 (0.7)	17
	MV	1.8 (0.5)	1.8 (0.5)	29	2.5 (0.7)	2.3 (0.5)	21
Chol/HDL ratio	PL	3.5 (0.9)	3.4 (0.8)	31	3.7 (0.7)	3.8 (0.8)	17 ^‡^
	MV	3.1 (0.6)	3.1 (0.6)	29	4.0 (0.9)	3.8 (0.7)	21
AST, *U/L*	PL	23.0 (5.7)	21.3 (5.3)	29 *	24.2 (6.0)	24.0 (7.1)	16
	MV	21.4 (45.0)	26.2 (9.7)	29	23.1 (6.2)	25.0 (5.9)	21
ALT, *U/L*	PL	22.9 (10.1)	20.9 (9.1)	29 *	27.5 (12.2)	27.6 (9.4)	16
	MV	18.1 (4.0)	22.9 (5.4)	27	25.6 (7.9)	30.5 (9.9)	21

MV = multivitamin, PL = placebo; * *p* < 0.001; ^†^ *p* < 0.01; ^‡^ *p* < 0.05 for the effect of treatment at follow up, controlling for baseline; CRP—high sensitivity C-reactive protein; AST—aspartate transaminase; ALT—alanine transaminase.

Treatment effects were observed for AST (*F*(1,56) = 22.84, *p* < 0.001, partial η^2^ = 0.290) and ALT (*F*(1,54) = 24.316, *p* < 0.001, partial η^2^ = 0.310) which both increased with supplementation. Although significant, the changes remained within the provided reference ranges: AST < 41 U/L and ALT < 51 U/L (Table 4).

### 3.3. Blood Test Results—Men’s Formula

Multivitamin supplementation increased vitamin B_6_ levels (*F*(1,25) = 23.6, *p* < 0.001 partial η^2^ = 0.486) and vitamin B_12_ levels (*F*(1,31) = 12.9, *p* = 0.001 partial η^2^ = 0.295) compared with placebo. There was a treatment effect for homocysteine (*F*(1,35) = 8.42, *p* = 0.006, partial η^2^ = 0.194), with a decrease in homocysteine in the multivitamin group and an increase in the placebo group. Paired samples *t*-tests indicated that the decrease in the multivitamin group approached significance (*t*(20) = 1.949, *p* = 0.066), whereas the increase in the placebo group was not significant (*p* = 0.308). There was no treatment effect for fibrinogen. There was no treatment effect for CRP, after logarithmic transformation.

There was a trend for a reduction in protein carbonyls after supplementation with the multivitamin compared with placebo (*F*(1,28) = 3.491, *p* = 0.072, partial η^2^ = 0.111). This reflected a 20% reduction in protein carbonyls in men taking the multivitamin compared with only a very small change in the placebo group. *Post-hoc t*-tests indicated that the change in the multivitamin group was significant (*t*(14) = 2.59, *p* = 0.021), whereas the change in the placebo group was not.

Supplementation with the Men’s formula had significant effects on some lipid measures. There was a significant treatment effect for LDL (*F*(1,35) = 5.14, *p* = 0.030, partial η^2^ = 0.131), and a marginal effect for total cholesterol (*F*(1,35) = 3.30, *p* = 0.078, partial η^2^ = 0.086). These changes were reflected in cholesterol ratios. The LDL/HDL ratio decreased only in the multivitamin group after supplementation (*F*(1,35) = 4.40, *p* = 0.043, partial η^2^ = 0.112). Similarly, there was a trend for a reduction in the cholesterol/HDL ratio (*F*(1,35) = 3.94, *p* = 0.055, partial η^2^ = 0.101). There were no significant changes in AST or ALT.

## 4. Discussion

The present investigation found no effect of 16-weeks multivitamin, mineral and herbal supplements on cognitive task performance in healthy women or men aged 55–65 years. Therefore, the hypothesis that these primary outcome measures would be improved with supplementation was not supported. However, beneficial changes to blood biomarkers were observed. In the Women’s group, vitamin B_6_ and vitamin B_12_ significantly increased after supplementation and the inflammatory marker CRP was reduced. Two markers of liver function, ALT and AST, were also increased however these increases were small and within levels of normal within-subject variation for these biomarkers [33]. In the Men’s group, blood levels of vitamin B_6_ and vitamin B_12_ were also significantly increased; homocysteine, total cholesterol and LDL were significantly reduced; and there was a trend for a reduction in protein carbonyl concentration, a measure of oxidative stress. ALT and AST were not significantly changed in the Men’s group.

The lack of effect observed for cognition is in contrast to two previous studies by our group, which found memory improvements in response to multivitamin supplementation, using the same computerized cognitive battery. These included one study examining the effects of a multivitamin, mineral and herbal combination on older men who were at increased risk of cognitive decline due to lifestyle factors including sedentary occupation [22], and another which examined effects in women aged 65 plus years with self-reported memory complaints [30]. This last study was in elderly women and used the same Women’s 50 plus multivitamin supplement that was used in the present Women’s study. A key difference in the present study is that participants were healthy and were not selected for characteristics that increase their risk of cognitive impairment. Furthermore, the previous studies included participants who were older than those in the Women’s and Men’s studies reported here. This is true for a further two studies of multivitamin supplementation in older adults, which found cognitive benefits [23,34]. Thus, the inclusion of participants with poorer health or older age may be an important factor in observing cognitive benefits due to supplementation.

Despite a lack of cognitive effects of the supplements in this study, there were beneficial changes to blood biomarkers. This is important because a reduction of risk biomarkers has the potential to protect against impairment over the longer term. In particular, inflammatory biomarkers, protein carbonyls and homocysteine are of interest. Cognitive decline is associated with increased chronic inflammation, oxidative stress and higher levels of homocysteine, and it has been argued that these risk markers are a cause of impairment and not just an indicator of it [28,35,36]. Changes to these biomarkers were observed differentially in the Women’s and Men’s studies. This may be due to gender differences in older adults, or to ingredients that were specific to each formula (Table 1). However, it is important to note that all changes were in beneficial directions, with the exception of the very small changes observed in ALT and AST in the Women’s group. Changes to ALT and AST are unlikely to be clinically significant, since they remained within the reference range; also measurement of these enzymes has been shown to be highly variable [33].

CRP is a marker of inflammation and has been shown to predict cognitive decline and dementia [37]. An effect of supplementation on CRP was observed in the Women’s group but not the Men’s group. This echoes findings from a cross-sectional study, which found that use of multivitamins was associated with lower CRP in women, whereas there was no such association in men [38]. The authors suggested that supplements may interact with gender-specific factors to produce a reduction in CRP. Gender differences are observed in population CRP levels; a recent report indicated that more women had elevated CRP than men (32% *vs.* 20%), however in women it was not associated with as high a risk of mortality [39].

A reduction in homocysteine was only observed in the Men’s group. The level of homocysteine reduction achieved in the Men’s study was a moderate 9.3%, substantially less than the 25% reduction that has previously been observed with B-vitamin supplementation [40], and less than the 12.5% reduction observed in our previous multivitamin study [22]. In the Vitacog study, participants with mild cognitive impairment supplemented for two years with B-vitamin supplementation and a 30% reduction in homocysteine was observed, with a concurrent slowing of cognitive decline in the treatment group; the higher dose and longer supplementation period might be necessary to observe changes [41]. Homocysteine is particularly sensitive to folate levels, folate being necessary for the conversion of homocysteine to methionine [28]. Therefore the poor response in the present study could be a result of folate malabsorption, which is not uncommon in older adults [42].

There was a 20% reduction in protein carbonyls in the Men’s group only. Although this effect only approached statistical significance, it is notable due to the size of the reduction. Protein carbonyls have previously been demonstrated to be sensitive to supplementation with combinations of vitamins including multivitamins [43,44]. A further study demonstrated protein carbonyl reduction in older adults after six months of vitamin E supplementation [19]. The change in protein carbonyls suggests that the antioxidant vitamins (or other herbal ingredients with antioxidant properties) reduced oxidative stress levels in the Men’s group only. Baseline protein carbonyl levels were higher in the men than in the women, perhaps allowing for a stronger effect of supplementation. Increased oxidative stress is thought to contribute to cognitive decline as well as neurodegenerative diseases such as Alzheimer’s disease [18]. Therefore a reduction in oxidative stress may improve cognitive outcomes in the elderly.

The Men’s study also observed improvements in the lipid profile. Improvements to the lipid profile have previously been observed after supplementation with antioxidant vitamins [45] and multivitamins [46]. A recent review examined the effects of dietary interventions and supplements on the lipid profile [47]. The authors concluded there was no evidence for an effect of vitamin C, chromium or tocotrienols in cholesterol reduction, however they determined that there was evidence for other food components, such as soy protein and phytosterols. Also, green tea, red wine, and red yeast rice extract were considered to be potentially effective. Although these phytonutrients were not included in the present supplements, the Women’s and Men’s formulas contained many plant extracts that also contain flavonoid compounds. However, research regarding supplements and the lipid profile is limited and further research is required to substantiate the present findings.

## 5. Conclusions

In summary, although the changes observed in the blood markers were small, they occurred over a relatively short time span and longer term benefits of supplementation may have a greater impact. Further research is therefore warranted to elucidate the role of nutritional supplements in cognitive aging. This study demonstrates the importance of assessing risk markers concurrently with cognitive tasks, because beneficial changes to risk factors may be crucial for cognitive protection during aging.
